# Kinetic and thermodynamic studies reveal chemokine homologues CC11 and CC24 with an almost identical tertiary structure have different folding pathways

**DOI:** 10.1186/s13628-017-0039-4

**Published:** 2017-09-12

**Authors:** Baosheng Ge, Xiaoyong Jiang, Yao Chen, Tingting Sun, Qiuxia Yang, Fang Huang

**Affiliations:** Center for Bioengineering and Biotechnology, China University of Petroleum (East China), Qingdao, 266580 People’s Republic of China

**Keywords:** Chemokine, Thermodynamics, Kinetics, Folding intermediate, Homologous protein

## Abstract

**Background:**

Proteins with low sequence identity but almost identical tertiary structure and function have been valuable to uncover the relationship between sequence, tertiary structure, folding mechanism and functions. Two homologous chemokines, CCL11 and CCL24, with low sequence identity but similar tertiary structure and function, provide an excellent model system for respective studies.

**Results:**

The kinetics and thermodynamics of the two homologous chemokines were systematically characterized. Despite their similar tertiary structures, CCL11 and CCL24 show different thermodynamic stability in guanidine hydrochloride titration, with D_50%_ = 2.20 M and 4.96 M, respectively. The kinetics curves clearly show two phases in the folding/unfolding processes of both CCL11 and CCL24, which suggests the existence of an intermediate state in their folding/unfolding processes. The folding pathway of both CCL11 and CCL24 could be well described using a folding model with an on-pathway folding intermediate. However, the folding kinetics and stability of the intermediate state of CCL11 and CCL24 are obviously different.

**Conclusion:**

Our results suggest homologous proteins with low sequence identity can display almost identical tertiary structure, but very different folding mechanisms, which applies to homologues in the chemokine protein family, extending the general applicability of the above observation.

**Electronic supplementary material:**

The online version of this article (10.1186/s13628-017-0039-4) contains supplementary material, which is available to authorized users.

## Background

In spite of the fact that great progress has been achieved in structural biology, it is still an outstanding puzzle how the sequence of a protein determines its tertiary structure, biological function and folding pathway [1, 2]. Previous work has already shown that a single mutation may greatly affect the structure, stability or function of a protein [3, 4], but some proteins significantly differ in sequence are found to share very similar structures and functions [1, 2]. To unravel this mystery, folding of homologous proteins has been compared [5, 6] with peripheral subunit binding domains [7, 8], homologs protein G & L [9], spectrin domain R15, R16 and R17 [1] and RNase family [10]. It is noticed that some homologues share very similar folding mechanism [5, 9], while some others significantly differ thermodynamically and kinetically [11–14]. To reveal the reason, more systematic study on the folding of homologous proteins is demanded.

CCL11 and CCL24 belong to the β chemokine or CC chemokine family [15, 16]. They are efficient chemotaxis attractants for lymphocytes and play important roles in allergic inflammation [17–20], therefore these proteins are promising area for therapeutic strategy [21–23]. Although CCL11 and CCL24 exhibit <40% sequence similarity, they share an almost identical tertiary structure, which contains an unstructured N terminus, a C-terminal α helix and three anti-parallel β sheets [23–25]. The disordered N-terminal region [26] together with a groove structure formed by β_2_ and β_3_ sheet regions [27, 28] are highly conserved and required in the binding and activation of receptors for chemotaxis [29]. Clinical studies [19, 30] as well as our previous work [31, 32] demonstrate that CCL11 and CCL24 have equal chemotaxis efficacy in vivo and similar binding affinity with CCR3 in vitro. These enable the two chemokines to be ideal homologous proteins to explore the relationship between structure, function and folding pathway. To our knowledge, there is no report yet on the folding study of chemokines.

Here, the thermodynamics and folding kinetics of the two homologous chemokines, CCL11 and CCL24, were systematically characterized and compared. Our results show that despite their similar three-dimensional structures and functions, there is an obvious difference in their thermodynamic stability and folding kinetics. The kinetics curves obtained on stopped-flow show that there are two phases in the folding/unfolding processes of CCL11 and CCL24, suggesting the existence of folding intermediate. Folding intermediates are commonly observed in the studies of protein folding. However, it is hard to determine whether the folding intermediate is on or off the folding pathway. In this work, by comparing the thermodynamic parameters obtained from kinetics and equilibrium experiments, we find that location of CCL11 and CCL24 folding intermediates can be well described using an on-pathway model.

## Results

### Circular dichroism (CD) analysis of CCL11 and CCL24

**Fig. 1:**
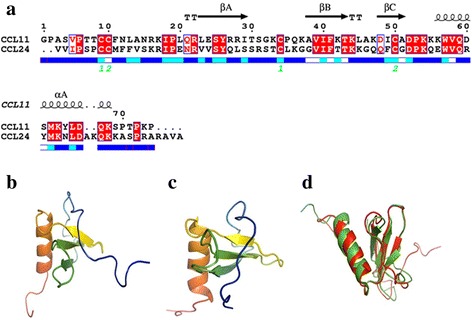
Sequence and **s**tructure comparison of CCL11 and CCL24. **a** sequence alignment of CCL11 and CCL24, **b** CCL11 (PDB ID: 1EOT), **c** CCL24 (PDB ID: 1EIG), and **d** the structure alignment of CCL11 (red) and CCL24 (green)

CCL11 and CCL24 are two typical chemokines having similar molecular weights of 8.4 kDa and 8.8 kDa, respectively. As typical chemokines, both CCL11 and CCL24 contain a disordered N-terminus, three β-sheets and one C-terminal α-helix. Amino acid sequence alignment shows that CCL11 and CCL24 exhibit only 39% amino acid sequence identity (Fig. 1a), however, previous studies indicate that they share an almost identical tertiary structures (Fig. 1b-d) and functions in vivo [20, 33], which enable them to be ideal homologous proteins for protein folding and unfolding studies.

**Fig. 2:**
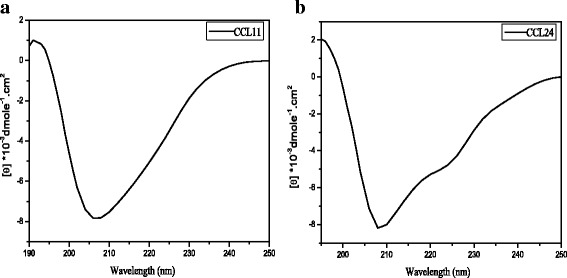
Circular dichroism spectrum of CCL11 (**a**) and CCL24 (**b**). The final spectrum was corrected for background by subtracting the corresponding buffer spectrum obtained under identical conditions. The mean residue ellipticity θ_MRE_ is calculated according to Eq. (2)

As shown in Fig. 2, the CD spectra of CCL11 and CCL24 do not show typical α-helix or β-sheet structure since both secondary structures exist in the proteins. CCL24 displays a weak negative peak at 222 nm and a strong negative peak at 208 nm, whereas CCL11 has a similar strong peak at 208 nm but a weaker negative peak at 222 nm. The stronger signal at 222 nm for CCL24 is likely due to its slightly longer α-helix structure than CCL11. Both the shape and amplitude of CD spectra for our prepared CCL11 and CCL24 are consistent with the previous reported data [31], suggesting that they have been properly folded to their native states.

### Equilibrium denaturation measurements of CCL11 and CCL24

**Fig. 3:**
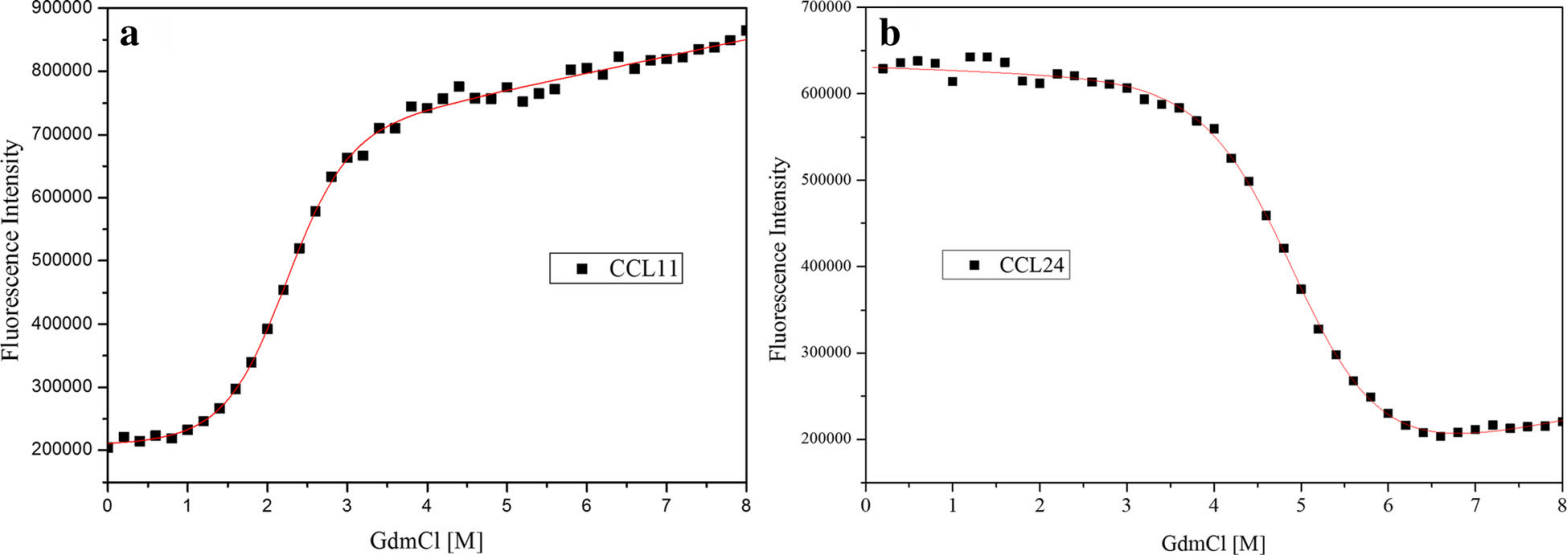
The chemical denaturation of CCL11 and CCL24 with guanidine hydrochloride. **a** the equilibrium titration curve of CCL11; **b** the equilibrium titration curve of CCL24. The protein denaturation curves were fitted using a two-state model (Eq. 3)

To gain deeper insight into the similarity and difference of CCL11 and CCL24, chemical stability of the two chemokines was determined using changes in intrinsic fluorescence as a function of the concentration of guanidine hydrochloride. As shown in Fig. 3, with the increase of guanidine hydrochloride concentration, the fluorescence intensity enhances gradually for CCL11 (Fig. 3a), whereas CCL24 shows an opposite tendency (Fig. 3b). This difference is likely due to the changes of local environment of tryptophan (Trp). Trp57 of the CCL11 locates between the β-sheet and α-helix. This region contains several polar amino acids, such as Lysine and Arginine, which could form a polar environment to quench the fluorescence of Trp57 [34]. Upon denaturation, this polar environment is broken, results in increasing of fluorescence intensity. Trp55 of the CCL24 locates inside the hydrophobic environment of α-helix, which is broken upon denaturation. Therefore Trp55 is gradually exposed to a polar environment and its fluorescence is quenched.

The equilibrium denaturation curves of CCL11 and CCL24 are well fitted to a simple two-state model (Fig. 3) and their results are illustrated in Table 1. In this study, it is noticed that CCL11 and CCL24 can be completely denatured in 4.5 M and 6.5 M guanidine hydrochloride, respectively (Fig. 3). As exhibited in Table 1, the D_50%_ and m-value are 2.20 M and 1.54 kcal mol^−1^ M^−1^ respectively for CCL11, and 4.96 M and 1.19 kcal mol^−1^ M^−1^ for CCL24, suggests that the native state of CCL24 has much higher thermodynamic stability than CCL11. According to m-values, the change in the solvent-accessible surface area (ΔSASA) of CCL11 seems to be larger than CCL24 upon denaturation.

**Table 1:** Thermodynamic stability parameters of CCL11 and CCL24^*a*^

	ΔG (kcal mol^−1^)	D_50%_ (M)	m (kcal mol^−1^ M^−1^)
CCL11	3.39 (±0.32)	2.20 (±0.04)	1.54 (±0.11)
CCL24	5.90 (±0.37)	4.96 (±0.03)	1.19 (±0.06)

^*a*^The standard errors were obtained from the fit, or of the mean, as appropriate

### Folding and unfolding kinetics of CCL11 and CCL24

**Fig. 4:**
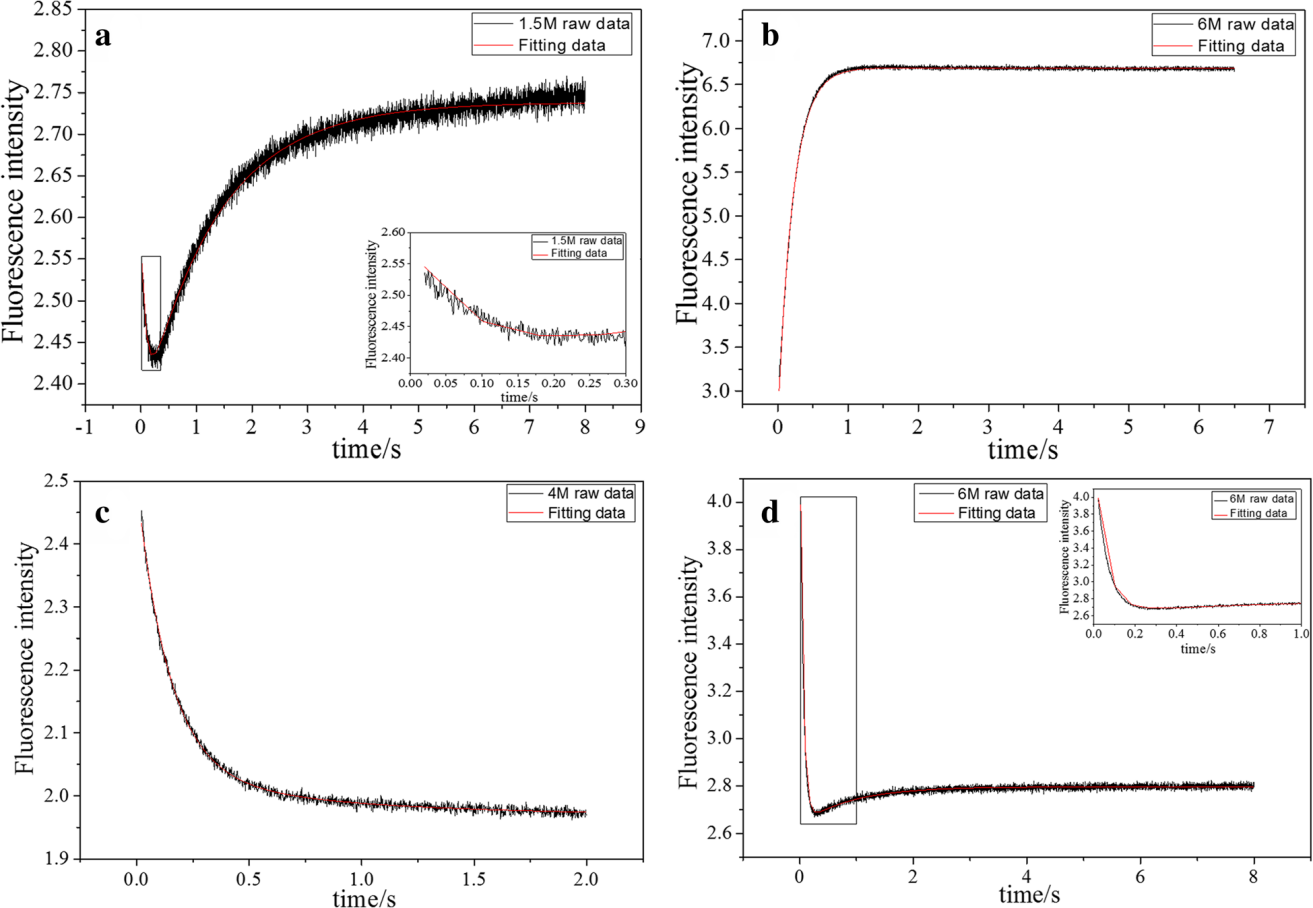
Folding and unfolding kinetics of the CCL11 and CCL24. **a** represents the folding traces of CCL11 with concentration of GdnHCl at 1.5 M. **b** represents the unfolding traces of CCL11 with concentration of GdnHCl at 6 M. **c** represents folding traces of CCL24 with concentration of GdnHCl at 4 M. **d** represents unfolding traces of CCL24 with concentration of GdnHCl at 6 M. The concentration of proteins was set as 100 μM in phosphate buffer. The fluorescence signal was recorded at 320 nm with excitation wavelength as 280 nm at 25 °C

Folding and unfolding kinetics of the CCL11 and CCL24 under different concentration of guanidine hydrochloride (GdnHCl) were measured using stopped-flow method. The kinetics data of CCL11 shows two phases when concentration of guanidine hydrochloride is in the range of 0–3.5 M (Fig. 4a), while the data can be fitted well with a single exponential equation when the concentration of guanidine hydrochloride ranges from 3.5 M to 7 M (Fig. 4b). In contrast, the kinetics data of CCL24 at lower concentration of guanidine hydrochloride (0–5.5 M) is well suited for single exponential equation (Fig. 4c), while the kinetic trance at higher concentration of guanidine hydrochloride ranging from 5.5 M to 7 M the kinetic trance cannot be fitted with a mono-exponential equation (Additional file 1: Figure S1), and double exponential relaxation is observed (Fig. 4d). These results suggest that in the folding of CCL11 and CCL24 there exists an intermediate, but their folding kinetics is quite different.

It has been reported that disulfide bonds in CC chemokines play an important role in stabilizing the protein structures and their functions [35, 36], ewe then attempted to characterize the role of disulfide bonds during chemokine folding/unfolding process by reducing disulfide bonds using DTT or remove them by site-directed mutagenesis. As a result, the whole tertiary structure of chemokines became unstable and very easily denatured when one of the disulfide bonds were detached. When the two disulfide bonds were removed or reduced by DTT, the whole protein structure collapsed and could not fold into their correct structure. These results suggest that the formation of disulfide bonds is a critical step during chemokine folding from the amino acid sequence into their functional tertiary structures.

**Fig. 5:**
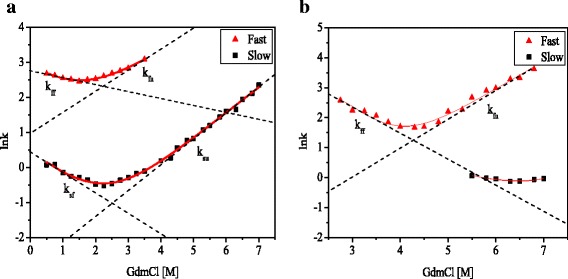
Chevron plots of CCL11 (**a**) and CCL24 (**b**). The plots were derived from stopped-flow experiments and fitted with Eq. 1

To fully characterize the two phases of CCL11 and CCL24 during folding/unfolding process, the Chevron plots derived from stopped-flow analysis over a wide range of guanidine hydrochloride conditions were fitted with Eq. 1 [37] and illustrated in Fig. 5.

1 $$ \mathrm{lnk}=\ln \left({k_f^{H_2O}}^{\ast}\exp \left(-{{\mathrm{m}}_f}^{\ast}\frac{x}{1.986^{\ast }298}\right)+{k_u^{H_2O}}^{\ast}\exp \left({{\mathrm{m}}_u}^{\ast}\frac{x}{1.986^{\ast }298}\right)\right) $$

where *k* is the observed relaxation rate constant, *x* is the concentration of denaturant, ${k}_f^{H_2O}$ and ${k}_u^{H_2O}$ are the constants for folding and unfolding in the absence of denaturant, respectively, m_*f*_ and m_*u*_ are the m values for folding and unfolding process, respectively.

From the Chevron plots (Fig. 5), it can be seen that for CCL11, the fast phase appears at low concentration of guanidine hydrochloride, while the slow phase appears in the entire range. In case of CCL24, the state is different, fast phase appears in the whole range of guanidine hydrochloride while the slow phase appears at high concentration of denaturant. These suggest that an intermediate state exists in their folding/unfolding process, but their stability are obviously different for CCL11 and CCL24 [38].

The folding kinetics data from stopped-flow can be well described by a three-state model with two folding steps. By fitting the data in Fig. 5, the folding and unfolding rate constant in the fast step named as *k*_ff_ and *k*_fu_, and those in the slow step as *k*_sf_ and *k*_su_, as well as their corresponding m values, are obtained and shown in Table 2. Based on these known folding and unfolding kinetics corresponding to the fast and slow phases, the change in free energy for each step were obtained (Table 2). It is noticed that the change in free energy for the fast and slow steps in the folding of CCL11 are −1.06 and −2.10 kcal mol^−1^, respectively, both of which are smaller than the ΔG obtained from equilibrium titration (−3.39 kcal mol^−1^). However, the total change of free energy for the fast and slow step is −3.16 kcal mol^−1^, similar to the ΔG obtained from equilibrium titration. This similarity supports a two-step folding model very well. For CCL24, the change of free energy for the fast step is −2.91 kcal mol^−1^, smaller than the ΔG obtained from equilibrium titration (−5.90 kcal mol^−1^). The data for the slow step of CCL24 span a narrow guanidine hydrochloride concentration and cannot be fitted accurately, due to the limited number of data points and the multiple parameters in the fitting equation. The kinetics in the absence of denaturant and corresponding thermodynamics is therefore not available.

**Table 2:** The fitted parameters from the CCL11 and CCL24 folding/unfolding kinetics data

	*k*_*ff*_(m_*ff*_)^a^	*k*_*fu*_(m_*fu*_)	ΔG_*fast*_(m_*fast*_^eq^)^a^	*p-value* ^△^	*k*_*sf*_(m_*sf*_)	*k*_*su*_(m_*su*_)	ΔG_*slow*_(m_*slow*_^eq^)	*p*-value^△^
CCL11	15.9 ± 0.6 (0.3 ± 0.1)	2.7 ± 0.8 (−0.4 ± 0.1)	−1.1 ± 0.1 (0.7 ± 0.02)	<0.01	2.4 ± 0.1 (0.4 ± 0.02)	0.1 ± 0.02 (−0.5 ± 0.01)	−2.1 ± 0.1 (0.9 ± 0.02)	<0.05
CCL24	17.4 ± 0.1 (0.2 ± 0.1)	0.1 ± 0.02 (−0.5 ± 0.01)	−2.9 ± 0.1 (0.7 ± 0.02)	<0.05	–	–	–	

^a^The units for *k*, m and ΔG are s^−1^, kcal mol^−1^ M^−1^ and kcal mol^−1^, respectively. The kinetic parameters (such as *k*_*ff*_ *, k*_*fu*_ *, k*_*sf*_ and *k*_*su*_) were fitted and averaged at least from three repeats. ^△^The *p*-values were obtained using t-test function of Excel software

## Discussion

Homologous proteins have been demonstrated to be useful models to answer the question how proteins find their unique native structures by using information encoded in their amino acid sequences. Several studies support that homologous proteins fold through similar folding pathways and transition states [2, 39], while other reports suggest that the folding rate of proteins with diverse sequences strongly correlated with more global parameters such as contact order, topology, average hydrophobicity, and protein length [40, 41]. Therefore, more homologous proteins should be compared to scrutinize the similarity and difference of folding pathways.

CCL11 and CCL24 exhibit only 39% amino acid sequence identity, however, they show almost identical tertiary structures and functions in vivo [20, 33], which enable them to be an ideal model for folding/unfolding studies of homologous proteins. In this work, the folding/unfolding kinetics and thermodynamics of CCL11 and CCL24 were systematically characterized, reveals that chemokine homologues, CCL11 and CCL24, show varied thermodynamic stability and diverse folding kinetics.

The kinetic experiments show clearly two-exponential decay, which suggests the existence of intermediates in their folding/unfolding process. However, it is also noticed that the equilibrium titration curves fit very well with a two-state folding model. Similar inconsistency between kinetics and equilibrium experiments is frequently observed in many previous reports [42–45]. These results indicate that the folding intermediate exists kinetically and compulsively on the folding pathway, so that it can be captured in the kinetic experiments. However, the folding intermediate cannot be accumulated thermodynamically under equilibrium due to its relatively high free energy.

The slow folding steps observed in protein folding are frequently designated to proline *cis-trans* isomerization [46, 47]. In this study, a slow step was observed in the folding/unfolding of both CCL11 and CCL24. For CCL11, the slow step exists in the whole range of denaturant concentration, while in CCL24 it only exists at high denaturant concentration. The slow step detected in this work is not assigned to proline *cis-trans* isomerization due to a few reasons. Firstly, a typical activation energies for proline isomerization is in the range of 18 and 22 kcal mol^−1^ [48], much higher than the estimated 2–4 kcal mol^−1^ observed here. Secondly, isomerization rates are generally in the range of 10–100 s, while the rate of slow step is in a few seconds. Finally, a clear “V” shape in the Chevron plot is observed in this study, while proline *cis-trans* isomerization is normally denaturant independently [48]. As such, it seems unlikely that proline isomerization was involved in the folding/unfolding of CCL11 and CCL24.

**Fig. 6:**
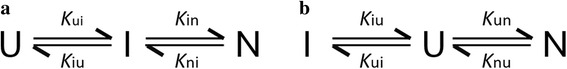
Possible three-state kinetic schemes for protein folding. U represents the unfolded ensemble; I represents the intermediate ensemble; N represents the native state. (**a**) on-pathway model, (**b**) off-pathway model

Folding intermediates are observed in the folding process of many proteins, but it is hard to determine whether the intermediate is on or off the reaction pathway and to characterize it [49, 50]. In this work, our data demonstrate the existence of folding intermediate. The next step is to determine where the folding intermediate is. As shown in Fig. 6, there are mainly two possibilities for a folding pathway with folding intermediate. Fig. 6 (a) shows a reaction with on-pathway folding intermediate and Fig. 6 (b) shows a reaction with off-pathway folding intermediate. It is possible to distinguish different mechanisms by comparing the thermodynamic parameters obtained from kinetic and equilibrium experiments. The free energy change for each step and for the whole folding process can be obtained from the kinetic experiments. Assuming that the intermediate is on the folding pathway, the change of total free energy for the whole folding process should be consistent with that obtained from equilibrium titration experiments. Otherwise, for an off-pathway folding, the free energy change obtained from equilibrium titration should be equal to that corresponding to U-N transition, i.e. one of the two folding steps in Fig. 6b. According to our fitting results, for CCL11 the free energy of each folding steps derived from kinetic experiments is different from the ΔG obtained from equilibrium titration, however, the total change in free energy for the fast and slow steps keeps good consistence with the ΔG obtained from equilibrium titration, which strongly suggests that CCL11 folds with an on-pathway folding intermediate. For CCL24, it is hard for us to accurately fit the slow unfolding step, and then the change of free energy for the slow steps is not available. According to its total change of free energy from equilibrium titration and the change of free energy obtained kinetically for the fast step, we supposed that CCL24 also folds with an on-pathway folding intermediate. It should be noted that one of the folding step disappears under certain conditions (Figs. 4 and 5). This suggests the folding intermediate for CCL24 is not stable at lower denaturant concentration, while that for CCL11 is not stable at higher denaturant concentration.

Despite the significant clinical application of chemokines, they tend to form inclusion bodies when expressed in *E. coli*, which need to be resolubilized using urea/guanidine hydrochloride and refolded in vitro for their structural and functional research [51, 52]. In this work, our studies on folding kinetics and thermodynamics of CCL11 and CCL24 describe systematically the change process of protein folding/unfolding states, and the percentage of denatured proteins in different concentration of guanidine hydrochloride, which would provide useful information on how to obtain high quality chemokines through refolding techniques.

## Conclusions

Thermodynamic and kinetic studies of CCL11 and CCL24 reveal that there is obvious difference in their thermodynamic stability and kinetics during their folding/unfolding processes. Although they both have an on-pathway intermediate state in the protein folding process, kinetics and location of the intermediate state are obviously different. Our results clearly support the opinion that not only does sequence encode the tertiary structure, which is robust to relatively large differences in primary structure, but the sequence also encodes the kinetics and thermodynamics of the folding process. Homologous proteins with low sequence identity can display identical tertiary structure, but different folding mechanisms. This notion also applies to homologues in the chemokine protein family.

## Methods

### Protein expression and purification

The amino acid sequences of mature CCL11 and CCL24 were obtained from NCBI database and corresponding genes were commercially synthesized by GenScript Bio Company (Nanjing, China) after codon optimization for overexpression in *Escherichia coli* (*E. coli*). The synthesized genes were then sub-cloned into pET28a plasmid (Novagen) and transformed into *E. coli* BL21 (DE3). The CCL11 and CCL24 proteins were overexpressed and purified as previously described [31]. The purified proteins were confirmed using SDS-PAGE and mass spectrum, and then stored at −80 °C for further use.

### Circular dichroism spectroscopy analysis

Circular dichroism (CD) experiments were performed on a MOS-450 circular dichroism spectrometer (Bio-Logic Science Instruments, France). The purified CCL11 and CCL24 proteins were buffer-exchanged to phosphate buffer (20 mM phosphate, pH 7.0). Far-UV CD spectrum was obtained from 190 nm to 250 nm with a 2 mm path length cell at 25 °C with protein concentration of 25 μM,where the step size and acquisition time were set as 1 nm and 5 s, respectively. The spectra were finally background-subtracted using corresponding buffer spectrum under the same conditions. Results were expressed as mean residue ellipticity θ_MRE_, calculated according to Eq. (2), where θ_obs_ is the observed ellipticity (in deg), *d* is the path length (in cm), *C* is the concentration of protein samples (M), *n* is the total number of amino acids in the protein.

2 $$ {\uptheta}_{MRE}=100\ast {\uptheta}_{obs}/\left[ dC\left(n-1\right)\right] $$

### Equilibrium titrations of CCL11 and CCL24

Chemical denaturation experiments were performed on a Fluorescence Spectrometer (FluoroMax-4, Horiba Jobin Yvon). The final concentration of guanidine hydrochloride solutions were set in the range of 0 M and 8.0 M with gradient interval of 0.2 M. Fluorescence spectra were recorded from 290 nm to 450 nm with excitation wavelength at 280 nm and slit size of 5 nm at 25 °C. The protein denaturation curves were fitted with Eq. (3) according to a two-state model [53].

3 $$ \mathrm{Y}=\frac{{\mathrm{a}}_N+{{\mathrm{b}}_N}^{\ast}\mathrm{x}+{\left({\mathrm{a}}_D+{{\mathrm{b}}_D}^{\ast}\mathrm{x}\right)}^{\ast}\exp \left({\left(\frac{1}{298}\right)}^{\ast }{\mathrm{m}}^{\ast}\frac{\mathrm{x}-{\mathrm{D}}_{50}}{1.986}\right)}{1+\exp \left({\left(\frac{1}{298}\right)}^{\ast }{\mathrm{m}}^{\ast}\frac{\mathrm{x}-{\mathrm{D}}_{50}}{1.986}\right)} $$

where a_*N*_ is the fluorescence intensity of the native state; a_*D*_ is the fluorescence intensity of the denatured state; b_*N*_ and b_*D*_ represent the slope of the baseline of the native and denatured side, respectively; D_50%_ is the denaturant concentration at which 50% of protein is unfolded; m is a constant of proportionality and reflects the change in solvent-accessible surface area (ΔSASA) between the unfolded and folded states.

### Folding and unfolding kinetics of CCL11 and CCL24

The folding and unfolding kinetics of the CCL11 and CCL24 in guanidine hydrochloride solution were characterized on a stopped-flow system (Bio-Logic SFM300, France). The concentration of CCL11 and CCL24 was set as 100 μM in phosphate buffer (20 mM phosphate, pH 7.0). The intrinsic fluorescence signal of CCL11 and CCL24 was recorded at 320 nm with the excitation wavelength as 280 nm at 25 °C. The dead time of instrument was determined as 3.4 ms. Folding process of CCL11 and CCL24 was initiated by mixing the denatured protein in high concentration of guanidine hydrochloride with corresponding buffer without denaturant. Unfolding process of CCL11 and CCL24 was initiated by mixing the native protein with different concentration of guanidine hydrochloride. The folding and unfolding kinetics traces at different concentration of guanidine hydrochloride were repeated for at least three times and then fitted using an exponential function.

## Additional file

Additional file 1: Figure S1. Comparison of folding and unfolding trances of CCL11 and CCL24 fitted with different exponentials. (A) Kinetics traces of CCL11 at 1.5 M GdnHCl fitted with monophasic relaxation eq. (B) Kinetics traces of CCL11 at 1.5 M GdnHCl fitted with biphasic relaxation eq. (C) Kinetics traces of CCL11 at 6 M GdnHCl fitted with monophasic relaxation eq. (D) Kinetics traces of CCL11 at 6 M GdnHCl fitted with biphasic relaxation eq. (E) Kinetics traces of CCL24 at 4 M GdnHCl fitted with monophasic relaxation eq. (F) Kinetics traces of CCL24 at 4 M GdnHCl fitted with biphasic relaxation eq. (G) Kinetics traces of CCL24 at 6 M GdnHCl fitted with monophasic relaxation eq. (H) Kinetics traces of CCL24 at 6 M GdnHCl fitted with biphasic relaxation equation. (a-h) represent the residues of the fits corresponding to (A-H) respectively. (DOCX 1613 kb)

## Abbreviations

ΔSASA: The change in the solvent-accessible surface area
CCL11: CC chemokine 11
CCL24: CC chemokine 24
CCR3: CC chemokine receptor 3
CD: Circular dichroism
*E. coli*: *Escherichia coli*
GdnHCl: Guanidine hydrochloride
Trp: tryptophan
ΔG: The changes of Gibbs free energy
