# Supplementary material for: Kinetic and thermodynamic studies reveal chemokine homologues CC11 and CC24 with an almost identical tertiary structure have different folding pathways
Source: BMC Biophys. 2017 Sep 12;10:7. doi: 10.1186/s13628-017-0039-4 (PMC5596964; doi:10.1186/s13628-017-0039-4)
Supplement: Additional file 1: Figure S1. — Comparison of folding and unfolding trances of CCL11 and CCL24 fitted with different exponentials. (A) Kinetics traces of CCL11 at 1.5 M GdnHCl fitted with monophasic relaxation eq. (B) Kinetics traces of CCL11 at 1.5 M GdnHCl fitted with biphasic relaxation eq. (C) Kinetics traces of CCL11 at 6 M GdnHCl fitted with monophasic relaxation eq. (D) Kinetics traces of CCL11 at 6 M GdnHCl fitted with biphasic relaxation eq. (E) Kinetics traces of CCL24 at 4 M GdnHCl fitted with monophasic relaxation eq. (F) Kinetics traces of CCL24 at 4 M GdnHCl fitted with biphasic relaxation eq. (G) Kinetics traces of CCL24 at 6 M GdnHCl fitted with monophasic relaxation eq. (H) Kinetics traces of CCL24 at 6 M GdnHCl fitted with biphasic relaxation equation. (a-h) represent the residues of the fits corresponding to (A-H) respectively. (DOCX 1613 kb) [file 13628_2017_39_MOESM1_ESM.docx]

Supplementary Figure S1

Fig. S1 Comparison of folding and unfolding trances of CCL11 and CCL24 fitted with different exponentials. (A) Kinetics traces of CCL11 at 1.5 M GdnHCl fitted with monophasic relaxation equation. (B) Kinetics traces of CCL11 at 1.5 M GdnHCl fitted with biphasic relaxation equation. (C) Kinetics traces of CCL11 at 6 M GdnHCl fitted with monophasic relaxation equation. (D) Kinetics traces of CCL11 at 6 M GdnHCl fitted with biphasic relaxation equation. (E) Kinetics traces of CCL24 at 4 M GdnHCl fitted with monophasic relaxation equation. (F) Kinetics traces of CCL24 at 4 M GdnHCl fitted with biphasic relaxation equation. (G) Kinetics traces of CCL24 at 6 M GdnHCl fitted with monophasic relaxation equation. (H) Kinetics traces of CCL24 at 6 M GdnHCl fitted with biphasic relaxation equation. (a-h) represent the residues of the fits corresponding to (A-H) respectively.

(a) represents the folding traces of CCL11 with concentration of GdnHCl at 1.5 M. (b) represents the unfolding traces of CCL11 with concentration of GdnHCl at 6 M. (c) represents folding traces of CCL24 with concentration of GdnHCl at 4 M. (d) represents unfolding traces of CCL24 with concentration of GdnHCl at 6 M. The concentration of proteins was set as 100 μM in phosphate buffer. The fluorescence signal was recorded at 320 nm with excitation wavelength as 280 nm at 25 ºC.
